# Exploring the well-being of community pharmacy professionals, turnover intention and patient safety: Time to include operational responsibility

**DOI:** 10.1177/17151635231152170

**Published:** 2023-02-15

**Authors:** Seyedehsan Etezad, Mark Fleming, Heidi A. Weigand, Christopher M. Hartt, Daniel J. Dutton, James R. Barker, Keith R. Brunt

**Affiliations:** Psychology Department, Saint Mary’s University; Psychology Department, Saint Mary’s University; Rowe School of Business; Department of Business & Social Sciences; Dalhousie Medicine New Brunswick, Faculty of Medicine, Dalhousie University, Halifax, Nova Scotia; Rowe School of Business; Dalhousie Medicine New Brunswick, Faculty of Medicine, Dalhousie University, Halifax, Nova Scotia; Faculty of Business, University of New Brunswick

## Abstract

**Background:**

The COVID-19 pandemic added significant occupational pressures on community pharmacists. The objective of this research project was to investigate the level of distress and burnout among community pharmacy professionals and its association with their retention within their occupation as well as patient safety outcomes.

**Method:**

We conducted a cross-sectional study on 722 community pharmacy professionals from all Canadian provinces using an online survey, including scientifically validated measures. The data were analyzed using multiple regression analysis.

**Results:**

In Canada, 85% of community pharmacy professionals reported their mental health had suffered since the COVID-19 pandemic. Younger pharmacy professionals and those paid hourly reported a worsening level of mental health and an increasing level of turnover intention. Pharmacists with more dynamic/disrupted work schedules and those working for a large pharmacy chain (more than 25 pharmacies in Canada) reported lower levels of mental health quality. Pharmacy professionals working in pharmacies that are open more than 70 hours a week reported a lower level of patient safety culture. Pharmacists’ mental health was the significant predictor of their turnover intention, implying a heightened risk to professional effectiveness and retention. Compassion satisfaction was positively associated with patient safety culture and safety behaviour, while compassion fatigue and secondary traumatic stress were significantly associated with pharmacists’ level of risk-taking behaviours.

**Conclusion:**

This study emphasized the importance of prioritizing the mental health and well-being of community pharmacy professionals and demonstrated individual and systemic factors predicting the well-being and turnover intention of community pharmacists, as well as patient safety culture within their pharmacy. This research makes a case to consider actions to shift the monitoring focus from community pharmacists (also known as “individual responsibility”) to community pharmacies (also known as “operational responsibility”) for managing patient safety. Additionally, community pharmacists should be provided with the professional autonomy to affect their working conditions and alleviate the stress that has the potential to negatively affect the delivery of care.

**Knowledge into Practice**

- In total, 85% of community pharmacy professionals in Canada reported their mental health had become worse during the COVID-19 pandemic.
- This study emphasizes the importance of “securing your own mask before assisting others” within our health care system, to ensure we have a sufficient, effective and healthy workforce to lead community pharmacies and provide essential services to our communities.
- Policy-makers, health care administrators and industry stakeholders must prioritize community pharmacy professionals’ mental health and well-being.
- Regulatory oversight focus needs to shift to the pharmacies rather than the licensed pharmacists to ensure that occupational conditions, such as workload, work patterns and the work environment, meet obligations for safe health care delivery for staff and patients in accordance with legislated requirements.

**Mise En Pratique Des Connaissances**

- Quatre-vingt-cinq pour cent des professionnels en pharmacie communautaire au Canada ont déclaré que leur santé mentale s’était détériorée au cours de la pandémie de COVID-19.
- Cette étude souligne l’importance d’« assurer sa propre sécurité avant d’aider les autres » au sein de notre système de soins de santé, afin de pouvoir disposer d’une main-d’œuvre suffisante, efficace et en bonne santé pour diriger les pharmacies communautaires et fournir des services essentiels à nos collectivités.
- Les décideurs politiques, les administrateurs des soins de santé et les intervenants de l’industrie doivent prioriser la santé mentale et le bien-être des professionnels en pharmacie communautaire.
- La surveillance réglementaire doit mettre l’accent sur les pharmacies plutôt que sur les pharmaciens agréés pour s’assurer que les conditions de travail, comme la charge de travail, les régimes de travail et l’environnement de travail, répondent aux obligations en matière de sécurité de la prestation des soins de santé pour le personnel et les patients, conformément aux exigences législatives.

## Introduction

The COVID-19 pandemic has been the most significant public health event of the present era. It stress-tested the resiliency and safety of the Canadian health care system at various systemic, operational and individual levels.^1^ Such added pressure exposed critical failures in various areas of our health care system and mandated that community pharmacists adopt a unique role in the preparation, preparedness and response within their pharmacy practice. While still managing medical and drug supplies, providing coverage and ensuring patient-oriented safe use of medical/drug products, community pharmacies also provided more services in disease prevention and infection control than was typical before the pandemic.^2^ A recent study showed that the number of patients seeking pharmacist care increased significantly during the pandemic, as people avoided other avenues of health care due to the fear of contracting the virus.^3^ Such behaviour could have elevated exposure to community pharmacies without adequate coordination of workplace adaptations and frontline support in the pharmacy operating structures. Community pharmacists’ unique position and service locations mean they are the most accessible frontline essential health care workers in every community. Community pharmacists are highly skilled at triaging conditions associated with mental health, quality error-free pharmacological care and preventative measures, such as vaccination, chronic comorbidity monitoring and medication reviews. In Canada, there are more than 11,000 community pharmacies (1 for every 3500 Canadians).^4^ This accessibility to highly skilled professionals has encouraged policy-makers to expand community pharmacists’ scope of practice from adjudicators of safe medication delivery to providers of diverse clinical services in collaboration with primary care physicians. Although there is little doubt that this expanded scope of service has been a change in a positive direction, a 2020 article found that 1 of every 4 pharmacists in Canada believed their work environment was not conducive to providing quality and safe clinical care.^5^ Additionally, a study in the United States showed that 3 of every 4 community pharmacists were experiencing occupational burnout as a result of their job demands and working conditions, based on data collected after the COVID-19 pandemic.^6^ Anecdotal evidence from the Canadian population has shown the negative impact of the pandemic on the well-being of community pharmacy professionals, raising concerns regarding their turnover intention and patient safety.^7,8^ The objective of this research project was to investigate the level of well-being among community pharmacy professionals and its association with their turnover intention as well as patient safety outcomes. Therefore, we developed a broad survey, including scientifically validated measures that tap into the important psychological elements for medical professionals and provide community pharmacy professionals with an opportunity to reflect on how the pandemic has affected their abilities to provide safe, effective and sustainable health care.

## Methods

### Study design and participant recruitment

This was a cross-sectional survey study across Canadian provinces. The survey was available to participants in both English and French. Community pharmacy professionals were recruited through an e-mail sent by their respective pharmacy association or college of pharmacy as well as community pharmacy groups on social media. The data were collected from August 2021 to April 2022. A final sample included responses from 722 community pharmacy professionals in Canada.

### Data collection instrument and data analysis

All respondents provided informed consent and their participation was completely voluntary. In terms of compensation, participants were entered into a drawing of ten $100 gift cards (participants’ e-mail addresses were collected via a separate survey to ensure anonymity). Participants had the option to choose “prefer not to answer” for each item. No personal or identifiable information was collected in the main survey, except the IP addresses to control for duplicate entries from the same user. This study was approved by the Research Ethics Board of Dalhousie (2021-5518) and Saint Mary’s University (21-106).

The online survey included demographic items (i.e., sex, age, marital status, ethnicity, having dependents), working conditions (i.e., job status, job compensation, work shift), pharmacy-related variables (e.g., open hours, location, size), pandemic-related variables (e.g., perceived risk, infection control, COVID-19 vaccine administration) and evidence-based measures assessing job satisfaction,^9^ psychological safety (Edmondson),^10^ supervisor support,^11^ peer support,^11^ moral distress,^12^ mental health (GHQ-12),^13^ compassion satisfaction (ProQOL),^14^ compassion fatigue (ProQOL),^14^ secondary traumatic stress (ProQOL),^14^ turnover intention,^15^ presenteeism,^16^ absenteeism,^11^ patient safety culture (AHRQ),^17^ safety behaviours^18^ and risk-taking behaviours.^18^ All the scales had an acceptable level of reliability and internal consistency within our sample (the Cronbach alpha ranged from 0.79 to 0.96). Scale description and sample item for key variables in this study are listed in Table 1.

**Table 1:** Scale description and sample item for key variables

Variable	No. of items	Scale	Sample item
Mental health	12	4 points	Felt constantly under strain?
Compassion satisfaction	10	5-point Likert	I get satisfaction from being able to help people.
Compassion fatigue	10	5-point Likert	I feel trapped by my job.
Secondary traumatic stress	10	5-point Likert	As a result of my job, I have intrusive, frightening thoughts.
Job satisfaction	1	5-point Likert	In general, how satisfied are you with your job?
Psychological safety	7	7-point Likert	Members of this team are able to bring up problems and tough issues.
Supervisor support	1	5-point Likert	Do you perceive your supervisor as helpful and supportive?
Peer support	1	5-point Likert	Do you perceive your coworkers as helpful and supportive?
Moral distress	1	10 points	Moral distress is a form of distress that occurs when you believe you know the ethically correct thing to do, but something or someone restricts your ability to pursue the right course of action. How much moral distress have you experienced in your job?
Turnover intention	3	5-point Likert	I plan on leaving my job within the next year.
Presenteeism	1	Continuous	In the past 3 months, how many days have you gone to work despite feeling that you really should have taken sick leave due to your state of health?
Absenteeism	1	Continuous	In the past 3 months, how many work hours have you been absent from a regularly scheduled workday (e.g., calling in sick, using a personal day)?
Patient safety culture	40	5-point Likert	Think of patient safety inclusively in terms of pharmacy services—dispensing the right medication accurately, while making sure patients are effectively counselled and providing optimal outcomes in clinical services (currently allowed in your jurisdiction). How do you rate this pharmacy on patient safety?
Safety behaviours	3	5-point Likert	If I see someone breaking safety procedures, I confront them.
Risk-taking behaviours	5	5-point Likert	I take shortcuts that involve little or no risk.

Collected data were analyzed using SPSS version 26. Descriptive statistics and correlations for all study variables are presented in Appendix 1 (available in the supplemental materials to this article.). Multiple regression analysis was used to explain the unique role of each predictor in explaining the variance in the outcome variable controlling for demographic (block 1), pharmacy-related (block 2) and pandemic-related variables (block 3). Variables were entered stepwise in their respective block to evaluate their unique association with the outcome variable by looking at the change in *R*^2^ (reported in the tables). Participants with missing data were excluded list-wise. Multivariate assumptions of normality and linearity and univariate/multivariate outliers were examined and addressed before data analysis.

The investigators invite all community pharmacy stakeholders to use these data iteratively for collaborative analyses. The data that support the findings of this study are available from the corresponding author upon reasonable request.

## Results

The results of regression analyses are indicated in Tables 2–4.

**Table 2:** Hierarchical regression models predicting mental and occupational health of community pharmacy professionals

		Mental health			Compassion satisfaction			Compassion fatigue			Secondary traumatic stress		
Variable		*b*	*SE B*	β	*b*	*SE B*	β	*b*	*SE B*	β	*b*	*SE B*	β
Block 1	Intercept	1.143	0.130		26.722	1.903		33.455	1.695		27.948	1.862	
	Sex	–0.105	0.057	–0.087	–0.127	0.825	–0.007	0.122	0.748	0.008	0.142	0.804	0.009
	Age	0.005	0.002	0.109*	0.155	0.032	0.246^‡^	–0.141	0.028	–0.250^‡^	–0.093	0.031	–0.161^†^
	Marital status	0.012	0.061	0.010	0.776	0.886	0.044	0.103	0.805	0.006	–0.267	0.875	–0.017
	Ethnicity	0.079	0.052	0.070	2.078	0.768	0.128^†^	–1.778	0.684	–0.122*	0.609	0.754	0.041
	Dependents	0.038	0.052	0.036	1.102	0.752	0.073	–0.204	0.678	–0.015	1.123	0.747	0.081
	Job status	0.002	0.060	0.001	–0.326	0.877	–0.019	1.267	0.776	0.082	–0.011	0.872	–0.001
	Job compensation	0.246	0.066	0.179^‡^	3.288	0.974	0.163^†^	–2.419	0.877	–0.134^†^	0.305	0.953	0.017
	Work shift	–0.135	0.051	–0.125^†^	–0.721	0.738	–0.047	1.613	0.666	0.116*	–0.096	0.732	–0.007
*R* ^2^				0.09			0.12			0.13			0.03
Block 2	Intercept	1.138	0.140		27.707	2.060		32.861	1.831		29.445	2.032	
	Open hours	0.041	0.060	0.039	–1.245	0.863	–0.082	0.584	0.795	0.043	–0.404	0.875	–0.029
	Location	0.070	0.056	0.061	0.063	0.804	0.004	0.305	0.739	0.020	–0.788	0.806	–0.052
	Independent	–0.012	0.076	–0.009	–0.701	1.100	–0.036	–0.114	0.989	–0.007	–1.445	1.093	–0.082
	Grocery	–0.049	0.071	–0.034	–0.469	1.026	–0.023	0.217	0.940	0.011	0.376	1.029	0.020
	Large	–0.154	0.071	–0.143*	–1.368	1.022	–0.089	1.319	0.929	0.095	–1.446	1.028	–0.102
*R* ^2^				0.11			0.13			0.14			0.04
Block 3	Intercept	1.081	0.216		20.364	3.142		0.114	0.293	0.019	28.721	3.145	
	Risk from patients	–0.020	0.023	–0.042	0.202	0.328	0.031	0.261	0.304	0.047	0.220	0.326	0.036
	Risk from colleagues	0.005	0.023	0.011	–0.600	0.341	–0.096	–0.348	0.335	–0.065	–0.229	0.342	–0.040
	Risk from workspace	0.018	0.026	0.042	0.923	0.379	0.154*	0.814	0.337	0.144	0.093	0.376	0.017
	Risk from policies	–0.077	0.026	–0.174^†^	–0.663	0.377	–0.106	–1.643	0.377	–0.231*	1.183	0.383	0.206^†^
	Infection control	0.116	0.029	0.209^‡^	2.193	0.416	0.279^‡^	0.859	0.963	0.043^‡^	–1.159	0.426	–0.159^†^
	COVID vaccine	–0.095	0.073	–0.061	0.574	1.053	0.026	0.114	0.293	0.019	0.281	1.070	0.014
*R* ^2^				0.21			0.23			0.24			0.14
Block 4	Intercept	0.762	0.202		12.206	2.818		40.776	2.487		30.711	3.244	
	Job satisfaction	0.185	0.020	0.440^‡^	3.232	0.282	0.537^‡^	–2.767	0.253	–0.509^‡^	–0.606	0.324	–0.108
	Psychological safety	0.033	0.022	0.072	0.882	0.309	0.136^†^	–0.808	0.275	–0.140^†^	–1.301	0.353	–0.217^‡^
	Supervisor support	0.001	0.020	0.002	–0.272	0.278	–0.046	0.216	0.248	0.041	0.568	0.316	0.104
	Peer support	0.035	0.023	0.072	0.258	0.319	0.037	–0.392	0.280	–0.062	–0.635	0.361	–0.097
	Moral distress	–0.018	0.010	–0.082	0.128	0.142	0.040	0.181	0.126	0.063	0.638	0.163	0.218^‡^
*R* ^2^				0.42			0.48			0.51			0.27
Adj. *R*^2^				0.39			0.45			0.48			0.22

Sex: 0 = male, 1 = female. Marital status: 0 = single, 1 = couple. Ethnicity: 0 = Caucasian/majority, 1 = minority. Dependents: 0 = did not have dependents, 1 = had dependents. Job status: 0 = casual or part-time, 1 = full-time. Job compensation: 0 = hourly or contract, 1 = salary. Work shift: 0 = stable, 1 = mixed shifts. Open hours: 0 = fewer than 70 hours a week, 1 = more than 70 hours a week. Location: 0 = urban, 1 = rural. Independent: 0 = not independent, 1 = single independent pharmacy. Grocery: 0 = others, 1 = grocery. Large: 0 = fewer than 25 pharmacies in Canada, 1 = more than 25 pharmacies in Canada. *p < .05. ^†^*p* < .01. ^‡^*p* < .001.

**Table 3:** Hierarchical regression models predicting withdrawal behaviours of community pharmacy professionals

		Turnover intention			Presenteeism			Absenteeism		
Variable		*b*	*SE B*	β	*b*	*SE B*	β	*b*	*SE B*	β
Block 1	Intercept	3.463	0.344		5.953	1.535		17.229	3.704	
	Sex	–0.004	0.145	–0.001	1.192	0.652	0.091	–0.380	1.565	–0.012
	Age	–0.016	0.006	–0.149^†^	–0.064	0.025	–0.137*	–0.190	0.061	–0.166^†^
	Marital status	–0.306	0.164	–0.096	–0.481	0.745	–0.035	–2.794	1.780	–0.083
	Ethnicity	0.109	0.139	0.038	–0.126	0.625	–0.010	2.064	1.500	0.068
	Dependents	–0.023	0.135	–0.008	0.062	0.612	0.005	2.376	1.466	0.084
	Job status	–0.223	0.160	–0.072	–0.718	0.724	–0.053	–2.129	1.732	–0.065
	Job compensation	–0.437	0.169	–0.128*	–0.883	0.767	–0.060	2.883	1.836	0.081
	Work shift	0.382	0.133	0.141^†^	0.131	0.598	0.011	–0.966	1.440	–0.034
*R* ^2^				0.09			0.04			0.05
Block 2	Intercept	3.351	0.367		6.327	1.637		19.307	3.993	
	Open hours	0.294	0.165	0.109	0.816	0.741	0.070	0.125	1.801	0.004
	Location	–0.160	0.145	–0.055	–1.608	0.653	–0.129*	–1.531	1.582	–0.051
	Independent	0.014	0.185	0.004	–0.153	0.836	–0.011	–3.850	2.027	–0.115
	Grocery	0.304	0.197	0.078	1.604	0.884	0.096	1.291	2.151	0.032
	Large	0.282	0.184	0.104	0.364	0.833	0.031	0.168	2.023	0.006
*R* ^2^				0.13			0.07			0.07
Block 3	Intercept	4.255	0.544		5.863	2.570		26.086	6.312	
	Risk from patients	–0.002	0.056	–0.002	0.289	0.269	0.059	0.531	0.654	0.045
	Risk from colleagues	–0.081	0.060	–0.074	–0.429	0.288	–0.092	–0.677	0.703	–0.059
	Risk from workspace	0.081	0.067	0.077	0.066	0.318	0.015	0.932	0.777	0.085
	Risk from policies	0.113	0.066	0.103	0.738	0.318	0.158*	–0.643	0.774	–0.056
	Infection control	–0.409	0.075	–0.291^‡^	–0.462	0.358	–0.076	–1.568	0.875	–0.106
	COVID vaccine	0.014	0.182	0.004	–0.622	0.864	–0.038	–3.914	2.118	–0.098
*R* ^2^				0.26			0.11			0.09
Block 4	Intercept	4.078	0.882		3.706	4.660		29.547	11.700	
	Mental health	–0.518	0.152	–0.209^†^	–1.368	0.804	–0.129	0.366	2.014	0.014
	Compassion satisfaction	–0.028	0.011	–0.155*	–0.001	0.059	–0.001	–0.061	0.147	–0.033
	Compassion fatigue	0.033	0.017	0.167*	0.088	0.088	0.104	–0.068	0.220	–0.033
	Secondary traumatic stress	0.007	0.010	0.038	0.020	0.053	0.024	–0.006	0.131	–0.003
*R* ^2^				0.43			0.15			0.10
Adj. *R*^2^				0.40			0.10			0.04

Sex: 0 = male, 1 = female. Marital status: 0 = single, 1 = couple. Ethnicity: 0 = Caucasian/majority, 1 = minority. Dependents: 0 = did not have dependents, 1 = had dependents. Job status: 0 = casual or part-time, 1 = full-time. Job compensation: 0 = hourly or contract, 1 = salary. Work shift: 0 = stable, 1 = mixed shifts. Open hours: 0 = fewer than 70 hours a week, 1 = more than 70 hours a week. Location: 0 = urban, 1 = rural. Independent: 0 = not independent, 1 = single independent pharmacy. Grocery: 0 = others, 1 = grocery. Large: 0 = have fewer than 25 pharmacies in Canada, 1 = have more than 25 pharmacies in Canada. *p < .05. ^†^*p* < .01. ^‡^*p* < .001.

**Table 4:** Hierarchical regression models predicting patient safety culture and safety behaviours within community pharmacies

		Patient safety culture			Safety behaviour			Risk–taking behaviour		
Variable		*b*	*SE B*	β	*b*	*SE B*	β	*b*	*SE B*	β
Block 1	Intercept	3.059	0.177		2.537	0.227		2.694	0.210	
	Sex	0.028	0.075	0.018	–0.113	0.096	–0.056	–0.321	0.089	–0.174^‡^
	Age	0.011	0.003	0.196^‡^	0.015	0.004	0.205^‡^	–0.015	0.003	–0.219^‡^
	Marital status	0.061	0.085	0.036	0.156	0.109	0.073	–0.110	0.100	–0.056
	Ethnicity	0.215	0.072	0.142^†^	0.409	0.092	0.211^‡^	–0.023	0.085	–0.013
	Dependents	0.032	0.070	0.022	–0.022	0.090	–0.012	0.073	0.083	0.044
	Job status	0.095	0.083	0.058	0.189	0.106	0.091	–0.026	0.098	–0.014
	Job compensation	0.294	0.088	0.164^†^	0.199	0.113	0.087	0.149	0.104	0.071
	Work shift	–0.182	0.069	–0.127^†^	–0.072	0.088	–0.039	0.110	0.082	0.066
*R* ^2^				0.11			0.11			0.09
Block 2	Intercept	3.078	0.187		2.419	0.246		2.814	0.228	
	Open hours	–0.327	0.085	–0.231^‡^	–0.007	0.111	–0.004	–0.061	0.102	–0.037
	Location	0.091	0.074	0.060	0.169	0.097	0.087	–0.097	0.090	–0.055
	Independent	0.016	0.095	0.010	0.030	0.125	0.014	–0.092	0.116	–0.047
	Grocery	–0.010	0.100	–0.005	0.133	0.131	0.051	–0.071	0.121	–0.030
	Large	0.015	0.095	0.010	–0.007	0.124	–0.004	–0.024	0.114	–0.015
*R* ^2^				0.16			0.12			0.10
Block 3	Intercept	2.304	0.262		1.584	0.383		2.725	0.343	
	Risk from patients	0.114	0.027	0.191^‡^	0.136	0.040	0.179^†^	–0.002	0.035	–0.003
	Risk from colleagues	–0.051	0.029	–0.089	–0.085	0.042	–0.117*	0.037	0.038	0.055
	Risk from workspace	0.037	0.032	0.068	0.064	0.047	0.090	–0.052	0.042	–0.080
	Risk from policies	–0.137	0.032	–0.240^‡^	–0.020	0.047	–0.027	0.153	0.042	0.230^‡^
	Infection control	0.229	0.036	0.309^‡^	0.116	0.053	0.123*	–0.147	0.047	–0.170^†^
	COVID vaccine	0.059	0.089	0.029	0.044	0.130	0.017	0.183	0.117	0.078
*R* ^2^				0.36			0.17			0.20
Block 4	Intercept	2.595	0.438		–0.136	0.666		0.289	0.579	
	Mental health	0.012	0.076	0.009	0.079	0.115	0.047	0.085	0.100	0.056
	Compassion satisfaction	0.021	0.006	0.223^‡^	0.050	0.008	0.415^‡^	0.010	0.007	0.091
	Compassion fatigue	–0.020	0.008	–0.195^†^	0.012	0.013	0.090	0.037	0.011	0.307^†^
	Secondary traumatic stress	–0.003	0.005	–0.030	0.005	0.007	0.036	0.030	0.007	0.257^‡^
*R* ^2^				0.49			0.27			0.34
Adj*.R*^2^				0.46			0.23			0.30

Sex: 0 = male, 1 = female. Marital status: 0 = single, 1 = couple. Ethnicity: 0 = Caucasian/majority, 1 = minority. Dependents: 0 = did not have dependents, 1 = had dependents. Job status: 0 = casual or part-time, 1 = full-time. Job compensation: 0 = hourly or contract, 1 = salary. Work shift: 0 = stable, 1 = mixed shifts. Open hours: 0 = fewer than 70 hours a week, 1 = more than 70 hours a week. Location: 0 = urban, 1 = rural. Independent: 0 = not independent, 1 = single independent pharmacy. Grocery: 0 = others, 1 = grocery. Large: 0 = have fewer than 25 pharmacies in Canada, 1 = have more than 25 pharmacies in Canada. *p < .05. ^†^*p* < .01. ^‡^*p* < .001.

### Participants

The sample of this study consisted of 90.9% pharmacists (11.6% were owners, 22.6% managers, 54.1% staff pharmacists, 2.6% relief pharmacists) and 9.1% pharmacy assistants (unlicensed/unregistered), pharmacy technicians (licensed/registered) and interns. The largest number of participants were from Ontario (51.6%), followed by Nova Scotia (17.2%), New Brunswick (10%) and Alberta (7.7%). Most of the participants were female (72%), Caucasian (65.1%), working full-time (74.3%) and coupled (77.6%), with an average age of 41.51 years (SD = 12.51). About half of the participants had dependents (49.6%) and were working with a mixed working schedule (56.8%). Only 19.7% of the participants were on salary, and the other ones reported different forms of compensation (i.e., hourly or contract). In terms of pharmacy operation, 32.9% of the participants were working in pharmacies in rural areas. A total of 1.6% of the participants were working in grocery/supermarket pharmacies and 43.4% of the participants were working in large pharmacy chains (i.e., having more than 25 pharmacies in Canada). Also, 59.3% of the participants were working in pharmacies that operate more than 70 hours a week. A total of 84.7% of the participants were working in a pharmacy that offered COVID-19 vaccine administration to the public.

In terms of occupational health and well-being, this study focused on general mental health, compassion satisfaction (pleasure you receive from your job), compassion fatigue (negative feelings of hopelessness and frustration in meeting job expectations) and secondary traumatic stress, as well as its connection with patient safety culture. Patient safety culture is the extent to which a community pharmacy promotes patient safety through the following subfactors: (1) physical space and environment, (2) teamwork, (3) staff training and skills, (4) communication openness, (5) patient counselling, (6) staffing, work pressure and pace, (7) communication about prescriptions across shifts, (8) communication about mistakes, (9) response to mistakes, (10) documenting mistakes and (11) organizational learning and continuous improvement.

### Occupational well-being

#### Job satisfaction

Job satisfaction had the strongest relationship with community pharmacy staff’s levels of reported mental health, compassion satisfaction and compassion fatigue. Staff with higher levels of job satisfaction reported higher levels of mental health and compassion satisfaction and lower level of compassion fatigue.

#### Psychological safety

Psychological safety was significantly associated with compassion satisfaction, compassion fatigue and secondary traumatic stress among community pharmacy staff. Participants working in pharmacies with a psychologically safe culture reported a higher level of compassion satisfaction and lower levels of compassion fatigue and secondary traumatic stress. Additionally, based on the correlation analysis, psychological safety was positively associated with patient safety culture and safety behaviours and negatively associated with risk-taking behaviours.

#### Social support

Although supervisor and peer support did not show a significant association with mental and occupational health variables based on the regression analyses, their bivariate correlations with mental and occupational health as well as patient safety culture were significant.

#### Moral distress

Participants were asked to rate how much they had experienced moral distress in their job based on the following definition: “Moral distress is a form of distress that occurs when you believe you know the ethically correct thing to do, but something or someone restricts your ability to pursue the right course of action.” Based on the results, moral distress was significantly associated with secondary traumatic stress among community pharmacy staff.

#### Mental and occupational health

Employees’ mental and occupational health was the significant predictor of their turnover intention. Compassion satisfaction was significantly related to patient safety culture and safety behaviour in community pharmacies. Compassion fatigue and secondary traumatic stress were significantly associated with employees’ rate of risk-taking behaviours in the workplace.

### COVID-19 pandemic

#### Perceived risk

Participants were asked to rate their level of concern regarding 4 factors (i.e., patients, colleagues, workspace and work policies) that could potentially increase their exposure to the COVID-19 virus. Based on the results, the perceived risk from work policies had a significant negative association with pharmacy staff’s mental and occupational health. Perceived risk from patients had a significant positive relationship with patient safety culture, signalling that the more concerned the respondents were about being exposed to the virus from their patients, the better patient safety culture was in place. Conversely, the respondents reported a lower level of patient safety culture when they were concerned about being exposed to the virus due to high-risk work policies.

#### Infection control

Employees’ perception of the level of infection control in their pharmacy was a significant predictor of their level of mental health, occupational well-being and turnover intention. More specifically, employees who reported a high level of infection control within their pharmacy reported higher levels of mental health, compassion satisfaction, patient safety culture and safety behaviour and lower levels of secondary traumatic stress, risk-taking behaviours and turnover intention. The higher amount of infection control activities was positively associated with compassion fatigue, suggesting a negative impact of this extra task resulting from the COVID-19 pandemic.

#### COVID-19 vaccine administration

There was no significant difference between participants who administered the COVID-19 vaccine in terms of their mental health, occupational well-being, turnover intention, safety behaviours or patient safety culture compared with those who worked in the pharmacies that did not offer this service.

### Pharmacy operation

#### Type of pharmacy

Staff working in pharmacies that were open more than 70 hours a week reported a lower level of patient safety culture compared with the pharmacies that operated for fewer than 70 hours a week.

#### Size

Pharmacy staff working at large pharmacy chains (more than 25 pharmacies) reported lower levels of mental health compared with the ones who worked for small or medium-sized pharmacy chains.

#### Location

Pharmacy staff working in urban areas reported a higher level of presenteeism (i.e., working when feeling unwell) compared with community pharmacy staff working in rural areas.

### Job characteristics

#### Job status

There was no significant difference in any variables between full-time and part-time community pharmacy staff.

#### Compensation

Pharmacy staff who received hourly pay reported lower levels of mental health and compassion satisfaction and higher levels of compassion fatigue and turnover intention compared with their colleagues who received a salary. Additionally, employees receiving hourly pay reported a lower level of patient safety culture compared with their salaried counterparts.

#### Work shift

Participants were asked to identify whether they had fixed work shifts (i.e., always mornings, always afternoons, always nights) or mixed work shifts. Community pharmacy staff with mixed working schedules reported lower levels of mental health and patient safety culture and higher levels of compassion fatigue and turnover intention.

### Demographics

#### Sex

There were 3 people in our sample who identified themselves as nonbinary. Due to the small size of this group in our sample, our analysis of sex included only the individuals self-identifying as male/man or female/woman; however, those identifying as nonbinary were included in all other measures. There was no sex-related effect difference in terms of reported mental and occupational health, turnover intention or patient safety culture. However, male/man-identified participants reported a higher level of risk-taking behaviours in their workplace.

#### Age

There was a significant negative association between age and mental and occupational health variables, suggesting that a younger generation of community pharmacy staff reported lower mental health perceptions and compassion satisfaction and were more susceptible to compassion fatigue and secondary traumatic stress. Additionally, the younger generation of community pharmacy professionals reported a higher level of turnover intention, presenteeism and absenteeism. Moreover, younger pharmacy professionals reported a lower level of patient safety culture, a lower rate of safety behaviour and a higher rate of risk-taking behaviours within their work environment.

#### Marital status

There was no significant difference between participants who were coupled (i.e., married or common-law) and those who were single (e.g., single, separated, divorced, widowed) in any variable of this study.

#### Ethnicity

In terms of ethnicity, racial minorities reported higher levels of compassion satisfaction and lower levels of compassion fatigue. There was no significant difference between majority and minorities in terms of mental health and turnover intention. Regarding safety variables, minorities reported higher levels of patient safety culture and safety behaviours.

#### Dependents

There was no significant difference in any variables of this study between the participants who had dependents and those who did not (i.e., children under 22 years old or adults who are financially and physically dependent upon you).

## Discussion

**Figure 1:**
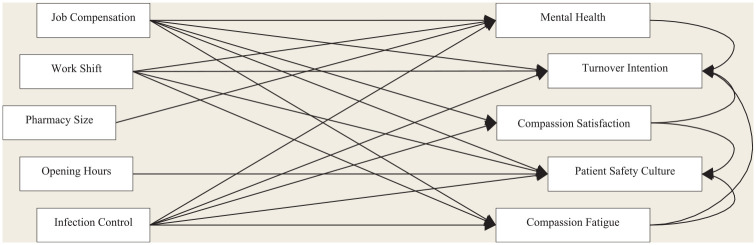
A conceptual summary of systematic variables predicting the well-being and turnover intention of community pharmacy professionals as well as patient safety culture within their pharmacy *Note*: Each path indicates a significant relationship between 2 variables in a hypothesized direction.

A conceptual summary of the key findings of this research project is presented in Figure 1. This study demonstrated a strong relationship between community pharmacy staff’s mental health and their turnover intention, as well as safety behaviours and patient safety culture. The findings support the previous limited and anecdotal evidence from news, social media and word of mouth suggesting a concerning level of well-being and turnover intention. Moreover, the impact on the younger professionals risks extending harm with pandemic resolution due to habituation, which could be exacerbated by premature workforce exit by experienced professionals. This would have substantive negative impacts across the community pharmacy sector from an industry perspective, as human resources and collective effectiveness will not be replaced by incumbent professionals. Based on the findings of this study, 85% of community pharmacy professionals in Canada reported their mental health had become worse than usual since the COVID-19 pandemic. Since the COVID-19 outbreak, despite introduction of a number of new policies and procedures on how to deliver care and services to patients, there has been no official guideline for community pharmacy professionals or owner/operators on how to engage with self-care activities and manage their own mental health and well-being, particularly with at-work policies/practices. To ensure we have an effective and healthy workforce to lead community pharmacies and provide essential services to our communities, policy-makers, health care administrators and industry stakeholders must prioritize the mental health and well-being of community pharmacy professionals. The findings of this study emphasize the importance of the airline adage of “securing your own mask before assisting others” within our health care system. Community pharmacists should be provided with the professional autonomy to affect their working conditions and alleviate the stress that might have been caused operationally (by pharmacy practices or corporations and government policies) or situationally (by unprecedented circumstances). Job stress as a risk factor has the potential to drive up the costs of care, reduce access to health care services and increase strain across the health care spectrum, including causing patient harm directly and indirectly. Based on the findings of this study, the worsening status of well-being among community pharmacy professionals, their high level of turnover intention and the poor patient safety culture present immediate and looming concerns that are not about individual pharmacists but are more about the system (the system that expects presenteeism and increases the workload without offering adequate staffing). Thus, a regulatory focus needs to be directed more toward the pharmacies rather than the pharmacists to help manage the conditions under which pharmacists operate. We recommend that government authorities shift their focus from monitoring “community pharmacists” to monitoring “community pharmacies” and hold pharmacy owners and corporations accountable for not providing a safe work environment for community pharmacy professionals, where detected. Equally, this accountability also requires payers (private and public) to participate proactively in the underlying provision of essential services equitably to meet legislated obligations and occupational expectations of the community pharmacy. This should be patient-centred but also requires an understanding of logistical needs underlying service delivery and continuity. This shift in agenda will potentially improve the well-being of community pharmacy professionals, which will consequently increase their retention within the profession as well as the patient safety culture within their pharmacy.

### Limitations

This was a cross-sectional survey study, meaning that no causal relationship can be interpreted. Although this study had a large sample size, the generalizability of the findings is somewhat limited since about half of the participants were from Ontario and we did not have an equally distributed sample from all the Canadian provinces and territories. Additionally, the spread of COVID-19 and the number of active cases changed rapidly during the data collection phase of this study, which means some participants in our sample may have responded to this survey when the number of active cases was low, whereas others had to deal with many active cases in their community. Most of our participants were recruited through our community partners (pharmacy associations or colleges of pharmacy). This may have created a sampling bias that might influence the generalizability of our findings, as some community pharmacy professionals are not members of pharmacy associations or they might have opted out of receiving research invitations from their respective colleges of pharmacy and were not included in our recruitment strategy. Although we tried to address this limitation through snowball sampling and social media recruitment, the sampling bias might still be present. Additionally, although we could not estimate how many community pharmacy professionals saw our advertisement, we believe the response rate was fairly low and the results are biased toward the ones who chose to participate in this study. However, because this study was focused on exploring the relationship between different variables rather than reporting their absolute values, we believe this limitation should not weaken the strong validity of our findings.

## Conclusion

Community pharmacy professionals are an integral part of our health care system and are legislated essential workers. If industry stakeholders and government authorities fail to support the well-being of pharmacists, they will consequently leave their profession which will endanger the quality and continuity of care within our health care system. This study indicated worsening well-being among community pharmacy professionals and a significant impact of this trend on their turnover intention and patient safety, with potentially lasting effects, particularly among younger pharmacy professionals, that could negatively impact the health care system and the pharmacy industry. ■

## Supplemental Material

sj-pdf-1-cph-10.1177_17151635231152170 – Supplemental material for Exploring the well-being of community pharmacy professionals, turnover intention, and patient safety: Time to include operational responsibility Click here for additional data file. Supplemental material, sj-pdf-1-cph-10.1177_17151635231152170 for Exploring the well-being of community pharmacy professionals, turnover intention, and patient safety: Time to include operational responsibility by Seyedehsan Etezad, Mark Fleming, Heidi A. Weigand, Christopher M. Hartt, Daniel J. Dutton, James R. Barker and Keith R. Brunt in Canadian Pharmacists Journal / Revue des Pharmaciens du Canada
