# Supplementary material for: Exploring the well-being of community pharmacy professionals, turnover intention and patient safety: Time to include operational responsibility
Source: Can Pharm J (Ott). 2023 Feb 15;156(2):71–84. doi: 10.1177/17151635231152170 (PMC10034527; doi:10.1177/17151635231152170)
Supplement: sj-pdf-1-cph-10.1177_17151635231152170 – Supplemental material for Exploring the well-being of community pharmacy professionals, turnover intention, and patient safety: Time to include operational responsibility [file sj-pdf-1-cph-10.1177_17151635231152170.pdf]

APPENDIX 1 Descriptive Statistics and Correlations for Study Variables

Variables	*M*	*SD*	1	2	3	4	5	6	7	8	9	10	11	12
1. G [a]	0.74	0.44	(-)											
2. A	40.88	12.10	-.032	(-)										
3. E [b]	0.32	0.47	-.167**	-.118*	(-)									
4. M [c]	0.76	0.43	-.006	.218**	.004	(-)								
5. D [d]	0.49	0.50	.017	.118*	.115*	.369**	(-)							
6. JS [e]	0.74	0.44	-.102	-.330**	.074	-.006	.004	(-)						
7. JC [f]	0.16	0.37	-.155**	-.070	-.001	-.006	.080	.215**	(-)					
8. WS [g]	0.59	0.49	.090	-.092	.036	-.066	-.022	.166**	-.176**	(-)				
9. OH [h]	0.51	0.50	-.026	-.077	.140**	-.021	.036	.111*	-.038	.451**	(-)			
10. PL [i]	0.29	0.45	.029	-.005	-.259**	.029	-.033	.002	-.010	-.240**	-.319**	(-)		
11. PI [j]	0.20	0.40	-.095	.052	-.043	.025	.007	-.157**	.059	-.222**	-.313**	.104*	(-)	
12. PG [k]	0.15	0.35	.039	-.042	-.003	-.069	.021	.021	-.102	.171**	-.023	-.014	-.187**	(-)
13. PL [l]	0.58	0.49	.019	.020	.059	-.046	.077	.082	-.220**	.390**	.482**	-.132*	-.580**	.309**
14. RP	3.97	1.17	.022	-.139**	.043	.013	.087	.070	-.115*	.132*	.167**	-.133*	-.034	.057
15. RC	3.27	1.24	.165**	-.062	-.020	.015	.105*	.039	-.102	.107*	.089	-.018	-.124*	.115*
16. RW	3.01	1.29	.108*	-.065	.011	-.029	.079	.006	-.051	.022	.160**	-.105*	-.043	.050
17. RPO	2.61	1.23	.082	-.081	.032	-.125*	.000	-.065	-.086	-.006	.100	-.028	.030	.021
18. IC	3.67	0.97	-.095	.067	.051	.036	-.019	.130*	.152**	-.052	-.172**	-.003	.035	.007
19. CV	0.86	0.34	.070	-.157**	-.036	-.022	.042	.160**	.026	.190**	.295**	-.006	-.223**	.144**
20. JSA	3.29	1.24	-.079	.178**	.085	.077	.089	-.029	.217**	-.173**	-.157**	.018	.116*	-.154**
21. PSY	4.81	1.17	.018	.227**	.029	.045	.023	-.032	.075	-.028	-.093	.062	-.010	-.037
22. SS	3.74	1.27	.005	.122*	.080	.019	-.001	.030	.120*	-.095	-.145**	.014	.009	.019
23. PS	3.97	1.05	-.047	.195**	.109*	.075	.070	-.017	.055	-.013	-.023	-.016	-.038	-.003
24. MD	3.84	2.41	.043	-.242**	-.023	-.088	-.021	.072	-.011	.061	.170**	-.053	.030	.011
25. MH	1.29	0.54	-.129*	.118*	.110*	.059	.079	.007	.208**	-.152**	-.053	.074	.083	-.110*
26. CS	35.05	7.60	-.058	.248**	.123*	.155**	.126*	-.041	.155**	-.116*	-.105*	.020	.072	-.075
27. CF	28.25	6.81	.050	-.260**	-.118*	-.076	-.071	.147**	-.139**	.186**	.117*	-.004	-.110*	.081
28. STS	24.71	6.98	.023	-.168**	.050	-.005	.044	.037	.014	.017	-.002	-.047	-.014	-.008
29. TI	2.60	1.35	.009	-.146**	.024	-.113*	-.055	-.040	-.159**	.133*	.178**	-.109*	-.077	.111*
30. PR	3.22	5.93	.099	-.125*	-.032	-.072	-.041	-.040	-.064	.016	.085	-.096	-.060	.099
31. AB	7.25	14.18	-.050	-.138**	.057	-.048	.063	-.003	.128*	-.091	.001	-.023	-.079	.037
32. PSC	3.65	0.71	-.063	.178**	.123*	.104*	.087	.001	.177**	-.134*	-.233**	.067	.076	-.021
33. SB	3.42	0.92	-.130*	.162**	.194**	.133*	.077	.066	.096	-.050	-.024	.048	.006	.015
34. SRT	1.88	0.83	-.155**	-.210**	.052	-.080	-.010	.115*	.095	.068	.047	-.052	-.011	-.004

Descriptive Statistics and Correlations for Study Variables

Variables	*M*	*SD*	1	2	3	4	5	6	7	8	9	10	11	12
Variables	13	14	15	16	17	18	19	20	21	22	23	24		
1. G [a]														
2. A														
3. E [b]														
4. M [c]														
5. D [d]														
6. JS [e]														
7. JC [f]														
8. WS [g]														
9. OH [h]														
10. PL [i]														
11. PI [j]														
12. PG [k]														
13. PL [l]	(-)													
14. RP	.102	(-)												
15. RC	.109*	.370**	(-)											
16. RW	.044	.373**	.560**	(-)										
17. RPO	-.031	.233**	.404**	.613**	(-)									
18. IC	-.046	-.215**	-.274**	-.422**	-.540**	(-)								
19. CV	.321**	.160**	.074	.066	.041	-.021	(-)							
20. JSA	-.218**	-.117*	-.187**	-.172**	-.255**	.382**	-.124*	(-)						
21. PSY	-.005	-.012	-.141**	-.176**	-.265**	.309**	-.048	.445**	(.83)					
22. SS	-.053	-.013	-.095	-.161**	-.297**	.482**	.000	.501**	.499**	(-)				
23. PS	-.009	-.064	-.133*	-.091	-.135**	.301**	.019	.337**	.519**	.411**	(-)			
24. MD	-.010	.241**	.263**	.346**	.392**	-.445**	.096	-.385**	-.381**	-.340**	-.177**	(-)		
25. MH	-.165**	-.164**	-.155**	-.152**	-.271**	.305**	-.122*	.587**	.333**	.334**	.273**	-.326**		
26. CS	-.148**	-.071	-.127*	-.089	-.226**	.315**	-.062	.637**	.380**	.355**	.304**	-.281**		
27. CF	.171**	.159**	.174**	.149**	.267**	-.304**	.150**	-.645**	-.419**	-.374**	-.306**	.383**		
28. STS	-.031	.126*	.121*	.194**	.270**	-.239**	.019	-.269**	-.359**	-.220**	-.228**	.378**		
29. TI	.176**	.131*	.132*	.241**	.277**	-.387**	.082	-.668**	-.445**	-.429**	-.253**	.378**		
30. PR	.094	.119*	.050	.139**	.204**	-.178**	.055	-.226**	-.182**	-.129*	-.102	.291**		
31. AB	.012	.058	.000	.072	.047	-.090	-.054	-.018	-.015	-.040	.055	.102		
32. PSC	-.129*	-.035	-.198**	-.237**	-.382**	.453**	-.064	.582**	.704**	.555**	.466**	-.430**		
33. SB	-.025	.102	-.067	-.003	-.085	.133*	-.005	.307**	.340**	.248**	.243**	-.091		
34. SRT	.004	.140**	.161**	.173**	.280**	-.226**	.075	-.189**	-.368**	-.185**	-.224**	.321**		

Descriptive Statistics and Correlations for Study Variables

Variables	25	26	27	28	29	30	31	32	33	34
1. G [a]										
2. A										
3. E [b]										
4. M [c]										
5. D [d]										
6. JS [e]										
7. JC [f]										
8. WS [g]										
9. OH [h]										
10. PL [i]										
11. PI [j]										
12. PG [k]										
13. PL [l]										
14. RP										
15. RC										
16. RW										
17. RPO										
18. IC										
19. CV										
20. JSA										
21. PSY										
22. SS										
23. PS										
24. MD										
25. MH	(.85)									
26. CS	.583**	(.93)								
27. CF	-.746**	-.752**	(.84)							
28. STS	-.504**	-.313**	.593**	(.87)						
29. TI	-.519**	-.482**	.528**	.321**	(.86)					
30. PR	-.291**	-.231**	.267**	.209**	.328**	(-)				
31. AB	.034	-.035	-.014	.027	.071	.283**	(-)			
32. PSC	.438**	.530**	-.544**	-.308**	-.527**	-.186**	.023	(.96)		
33. SB	.237**	.431**	-.288**	-.078	-.199**	-.129*	-.002	.468**	(.79)	
34. SRT	-.297**	-.235**	.424**	.462**	.266**	.197**	-.008	-.388**	-.197**	(.89)

G = gender, A = Age, E = Ethnicity, M = Marital Status, D = Dependents, JS = Job Status, JC = Job Compensation, WS = Work Shift, OH = Open Hours, PL = Pharmacy Location, PI = Pharmacy - Independent, PG = Pharmacy – Grocery, PL = Pharmacy – Large, RP = Risk from Patients, RC = Risk from Colleagues, RW = Risk from Work Space, RPO = Risk from Policies, IC = Infection Control, CV = COVID Vaccine, JSA = Job Satisfaction, PSY = Psychological Safety, SS = Supervisor Support, PS = Peer Support, MD = Moral Distress, MH = Mental Health, CS = Compassion Satisfaction, CF = Compassion Fatigue, STS = Secondary

Descriptive Statistics and Correlations for Study Variables

Traumatic Stress, TI = Turnover Intention, PR = Presenteeism, AB = Absenteeism, PSC = Patient Safety Culture, SB = Safety Behaviour, SRT = Safety Risk Taking Behaviour.

[a] 0 = male, 1 = female. [b] 0 = Caucasian/Majority, 1 = Minority. [c] 0 = single, 1 = couple. [d] 0 = did not have dependents, 1 = had dependents. [e] 0 = Casual or Part-time, 1 = Full-time. [f] 0 = hourly or contract, 1 = Salary. [g] 0 = Stable, 1 = Mixed Shifts. [h] 0 = Less than 70 hours a week, 1 = More than 70 hours a week. [i] 0 = Urban, 1 = Rural. [j] 0 = not independent, 1 = Single Independent Pharmacy. [k] 0 = Others, 1 = Grocery. [l] 0 = have less than 25 pharmacies in Canada, 1 = have more than 25 pharmacies in Canada. * $p < .05$, ** $p < .01$, *** $p < .001$.

Etezad S, et al. Exploring the well-being of community pharmacy professionals, turnover intention, and patient safety: time to include operational responsibility. Can Pharm J (Ott) 2023;156. DOI: 10.1177/17151635221152170.
